# Association between preoperative inflammatory status via CALLY index and postoperative pneumonia occurrence in resectable esophageal squamous cell carcinoma patients: a retrospective cohort study

**DOI:** 10.3389/fonc.2025.1486983

**Published:** 2025-02-17

**Authors:** Mengtao Fan, Yihan Zhu, Long Qian, Chuanxian Hu, Hui Ding

**Affiliations:** Department of Cardiothoracic Surgery, The Affiliated Huaian No. 1 People’s Hospital of Nanjing Medical University, Huaian, China

**Keywords:** esophageal squamous cell carcinoma, CALLY index, postoperative pneumonia, predictive marker, McKeown procedure

## Abstract

**Background:**

Postoperative pneumonia significantly affects recovery and prognosis in patients with esophageal squamous cell carcinoma. The CALLY index, derived from preoperative hematological parameters, may serve as a predictive marker for such complications.

**Objectives:**

To assess the association between preoperative inflammatory status via the CALLY index and the occurrence of postoperative pneumonia in patients with resectable ESCC.

**Methods:**

A retrospective cohort study was conducted from January 2020 to December 2022 at The Affiliated Huai’an No. 1 People’s Hospital of Nanjing Medical University. A total of 215 patients who met inclusion criteria were analyzed. Clinical data, including CALLY indices calculated preoperatively, were collected. Propensity score matching was applied to minimize confounding biases. The predictive value of the CALLY index was assessed using receiver operating characteristic analysis, and logistic regression was used to identify factors associated with postoperative pneumonia.

**Results:**

ROC curve analysis demonstrated the CALLY index had an area under the curve of 0.764 for predicting postoperative pneumonia, with a cutoff value of 1.97 achieving 67.69% sensitivity and 84.67% specificity. In multivariate analysis, a lower CALLY index was significantly associated with increased pneumonia risk, independent of other factors (adjusted OR = 0.66, p < 0.001). High CALLY index scores correlated with a decreased likelihood of postoperative pneumonia, reinforcing its utility as a non-invasive prognostic marker.

**Conclusions:**

The CALLY index is a robust, independent predictor of postoperative pneumonia in patients with resectable ESCC. Preoperative assessment of this index could enhance risk stratification and guide proactive management strategies to improve postoperative outcomes.

## 1 Introduction

Globally, esophageal cancer presents a formidable public health challenge, with the rates of occurrence and fatality exhibiting striking contrasts across distinct geographical locales (1, 2). Among its subtypes, esophageal squamous cell carcinoma (ESCC) represents a substantial fraction, with a pronounced prevalence in Eastern Asia and select African regions, influenced by region-specific environmental and lifestyle determinants (3, 4). Within China, the distribution of ESCC showcases pronounced regional disparities, being more frequent in the areas known as the “esophageal cancer belt,” which underscores the imperative for locally adjusted data analysis and strategic interventions (5, 6). For patients with resectable disease, surgical excision stands as the cornerstone of treatment, optionally complemented by neoadjuvant therapies, tailored according to the disease’s progression and individual patient factors (7–9). Nonetheless, the risk of post-surgical adversities—including arrhythmias, anastomotic leakages, and notably, pulmonary infections—remains a critical influencer of convalescence and long-term survival prospects (9–11).

Emerging research underscores the adverse impact of postoperative pulmonary infections in patients with esophageal squamous cell carcinoma (ESCC), noting their role in prolonging hospitalization durations and exacerbating healthcare expenditures (12–14). Additionally, such infections are correlated with diminished disease-free survival (DFS) and overall survival (OS), further complicating the clinical course (15–17). Consequently, timely and preemptive management is crucial for patients with ESCC who are predisposed to postoperative pneumonia. Typically, the recognition of postoperative pneumonia is contingent upon the emergence of symptomatic manifestations like a continual cough and fever, with radiological evidence from CT imaging providing confirmation of the infection, often at an advanced stage (18, 19). At such junctures, although interventions such as bronchoscopic sputum extraction and modifications to antibiotic protocols are available, the window for optimal therapeutic intervention may have narrowed (20). Hence, the prognostication of postoperative pneumonia via predictive markers discernible from perioperative clinical information is imperative to enhance patient management and outcomes.

Esophageal Squamous Cell Carcinoma (ESCC) represents a significant challenge to public health systems globally, with postoperative pneumonia being a key concern that compromises patient recovery and prognosis (21). While clinical and pathological staging has been the cornerstone of ESCC prognosis, novel preoperative hematological indices are now providing additional prognostic capabilities. These include counts of neutrophils, lymphocytes, monocytes, platelets, as well as serum albumin and C-reactive protein (CRP) levels, from which various ratios like the Neutrophil-to-Lymphocyte Ratio (NLR), Platelet-to-Lymphocyte Ratio (PLR), and Lymphocyte-to-Monocyte Ratio (LMR) are derived, showing promise in predicting surgical outcomes and complications (22–24). Notable advancements have been made in understanding the prognostic implications of these markers in solid tumors. Research by Ouyang et al. has brought to light the association between low pre-treatment NLR, reductions in NLR post-treatment, and favorable responses in metastatic colorectal cancer patients receiving immunotherapy (25). Lu et al. have highlighted the potential of PLR as a predictor of survival in patients with advanced hepatocellular carcinoma undergoing combination immunotherapy and kinase inhibitor treatments (26). Furthermore, Susiarno et al. demonstrated the effectiveness of PLR in distinguishing between benign and malignant ovarian tumors (27). Such findings affirm the prognostic value of NLR and PLR across various cancers, including ESCC (28). The CALLY Index, integrating albumin, lymphocyte count, and CRP, stands out as an emerging marker with demonstrated prognostic importance in several cancers like gastric, colorectal, and Non-Small Cell Lung Cancer (NSCLC) (29–31). It offers an all-encompassing measure that reflects a patient’s nutritional state, immune response, and inflammatory condition, thereby presenting a multifaceted view of their prognosis. However, the application of the CALLY Index in ESCC, especially in terms of forecasting postoperative pneumonia risk, is not thoroughly investigated, signaling a gap in the context-specific utility of this novel prognostic index.

In this study, we aimed to explore the role of the CALLY index, a composite score reflecting inflammatory and immune status, as a potential predictor of postoperative respiratory complications in patients undergoing surgery for esophageal squamous cell carcinoma. By employing a retrospective cohort approach, we investigated the association between preoperative CALLY index levels and the incidence of postoperative pneumonia, factoring in various clinical and demographic characteristics. This investigation seeks to contribute to the growing body of evidence regarding the prognostic value of hematological indices in surgical oncology, with the goal of enhancing preoperative risk assessment and informing tailored perioperative management strategies. Our study leverages multivariate logistic regression analysis to adjust for potential confounders and offers insights into the complex interplay between systemic inflammation and surgical outcomes.

## 2 Methods

### 2.1 Study design and participant selection

**Figure 1:**
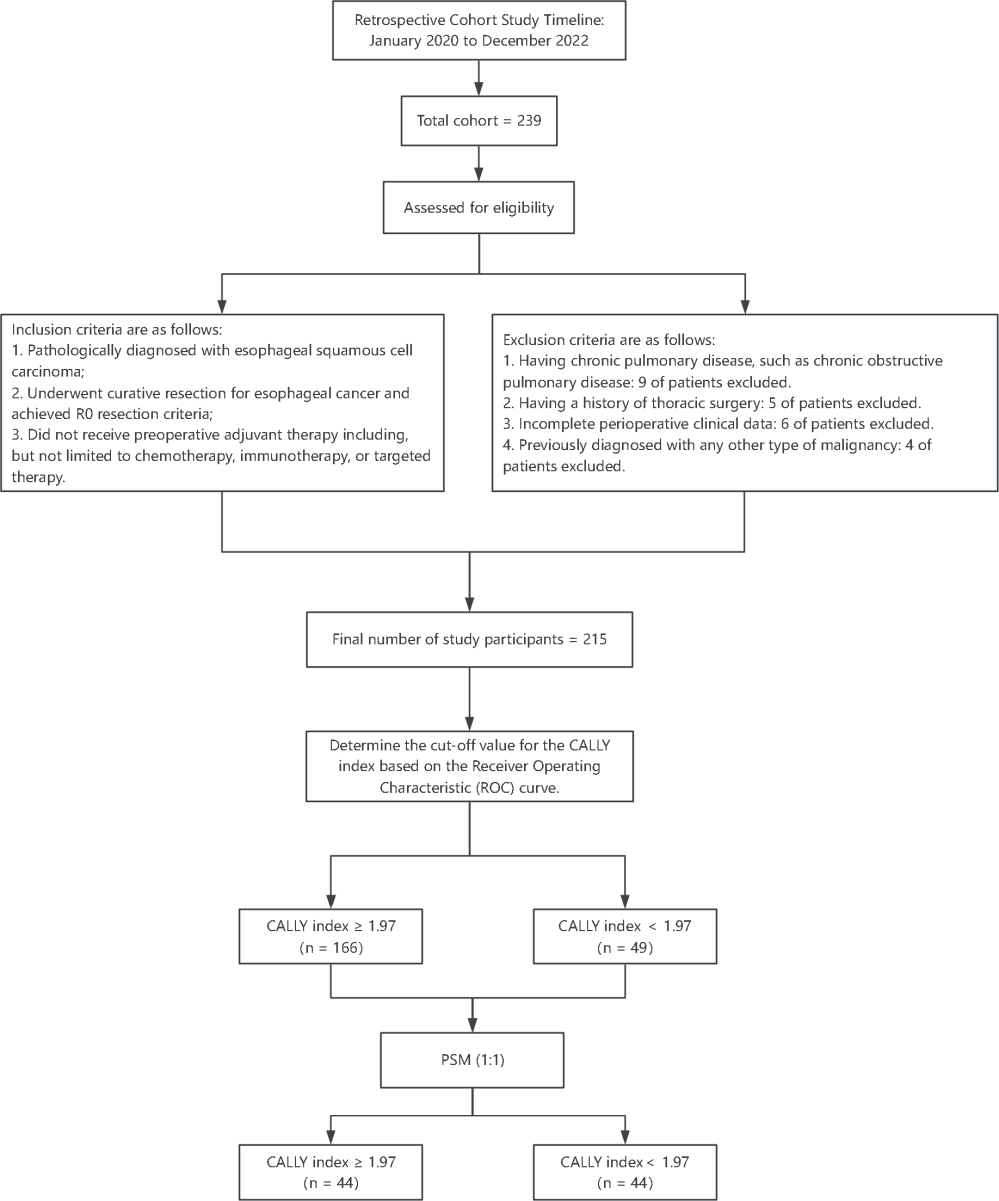
Study flowchart and participant selection. This flowchart outlines the retrospective cohort study design conducted from January 2020 to December 2022. Among an initial cohort of 239 patients with esophageal squamous cell carcinoma (ESCC), 215 eligible patients were selected based on inclusion criteria and exclusion criteria.

We conducted a retrospective cohort study in the Department of Cardiothoracic Surgery at The Affiliated Huai’an No. 1 People’s Hospital of Nanjing Medical University, covering the period from January 2020 to December 2022, as illustrated in **Figure 1**. This investigation aimed to evaluate the association between preoperative inflammatory status indicated by the CALLY index and the incidence of postoperative pneumonia among patients with resectable esophageal squamous cell carcinoma (ESCC) who underwent the thoraco-laparoscopic McKeown procedure. Individuals will be assessed for eligibility based on specific criteria prior to enrollment in the study. For inclusion in the research, participants must satisfy the following conditions: (i) histologically confirmed diagnosis of esophageal squamous cell carcinoma; (ii) completion of an esophagectomy aimed at curative intent with the attainment of clear margins (R0); (iii) absence of any preoperative neoadjuvant treatments which encompasses therapies such as but not restricted to chemotherapy, immunotherapy, or targeted therapy. In contrast, the criteria disqualifying prospective participants incorporate: (i) existence of any enduring pulmonary conditions, chronic obstructive pulmonary disease being an example; (ii) a medical record of any thoracic surgical procedures previously performed; (iii) lack of a comprehensive set of perioperative clinical records; (iv) a medical history indicative of another form of cancer. A total of 215 participants met these stringent criteria and were methodically staged according to the eighth edition of the TNM classification. Our meticulous methodological approach entailed obtaining informed consent from each subject prior to surgical intervention. The study was conducted in accordance with the principles outlined in the Declaration of Helsinki and received ethical endorsement from the Ethics Committee of Nanjing Medical University (approval no. KY-2022-008-01).

### 2.2 Collection of clinical data

In this retrospective cohort analysis, we collated a comprehensive set of potential clinical predictors. This included demographic information such as age and gender, along with pathological characteristics including tumor stage, which was determined based on established staging guidelines. The preoperative nutritional condition was assessed by measuring serum albumin levels prior to the surgical procedure. Additionally, we detailed observations relating to the recovery period post-surgery, with an emphasis on any complications that may have arisen during this period. For the operational definition of postoperative pneumonia, we adhered to rigorous criteria within the first 30 days following the surgical procedure. A diagnosis of pneumonia requires the fulfillment of three criteria: (i) confirmation through at least two thoracic imaging studies consistent with pneumonia, (ii) the presence of one or more clinical signs, including deviations in white blood cell count outside the normal range (either below 4 x 10^9/L or above 12 x 10^9/L), fever exceeding 38°C accompanied by changes in mental status, and (iii) the manifestation of at least two respiratory infection symptoms, which may include a new or altered sputum character, increased sputum production, a heightened need for secretion suctioning, or worsening shortness of breath.

### 2.3 Assessment of the CALLY index

Prior to surgery, an assessment of the preoperative inflammatory and immune profiles of the patients was performed through the measurement of various hematological parameters, specifically albumin (Alb), lymphocyte count, and C-reactive protein (CRP). These measurements were taken consistently within one week before the operation. The calculation of the CALLY index score involved the following formula: CALLY index = (Alb concentration in g/dL × number of lymphocytes per μL) divided by (CRP concentration in mg/dL × 10,000). This index facilitated an organized appraisal of the pre-surgical inflammatory and immune condition of the patient populace. To ensure uniformity and precision, all evaluations of the CALLY index were executed in a single, accredited laboratory that maintained strict adherence to established analytical procedures.

### 2.4 Statistical analysis

In our statistical analysis approach, the baseline characteristics of the study cohort were summarized using descriptive statistical methods. For continuous variables, the normality was assessed using Shapiro-Wilk test. Variables following a normal distribution were expressed as means with standard deviations, whereas non-normally distributed variables were described using medians and interquartile ranges. Categorical data were presented as frequencies and percentages. Statistical comparisons between groups involved the Chi-square test, independent samples t-test, and Mann-Whitney U test, ensuring each data type was appropriately evaluated. To address potential confounding and enhance comparability between high and low CALLY index groups, propensity score matching (PSM) was employed. PSM aimed to minimize selection bias by balancing covariates across comparison groups, thus ensuring that any differences in outcomes are more likely attributable to the CALLY index itself. This was executed using a 1:1 nearest neighbor matching algorithm without replacement, defining a caliper width of 0.2 standard deviations of the logit of the propensity score. The accuracy and optimal cutoff of the CALLY index as a prognostic tool were determined using receiver operating characteristic (ROC) curve analysis. Logistic regression curve fitting was utilized to model the association between CALLY index scores and the probability of postoperative pneumonia, offering insights into the relationship’s dynamics. A curve fitting analysis was further applied to evaluate non-linear relationships by modeling the effects of both the CALLY index and other clinical factors, such as the length of hospital stay on postoperative pneumonia risk, refining our understanding through exploratory non-linear modeling. Multivariate logistic regression analysis identified independent predictors of postoperative pneumonia, factoring in covariates selected based on clinical relevance and univariate analysis results. Statistical computations were executed in R software (version 4.1.0), employing packages such as dplyr for data manipulation, ggplot2 for data visualization, MatchIt for propensity score matching, pROC for ROC curve analysis, and MASS for logistic regression. All statistical procedures, including PSM and curve fitting, were performed using both SPSS (version 26.0) and R (version 4.1.0), with significance established at a p-value of less than 0.05.

## 3 Results

### 3.1 Prognostic utility of the CALLY index for postoperative pneumonia

**Figure 2:**
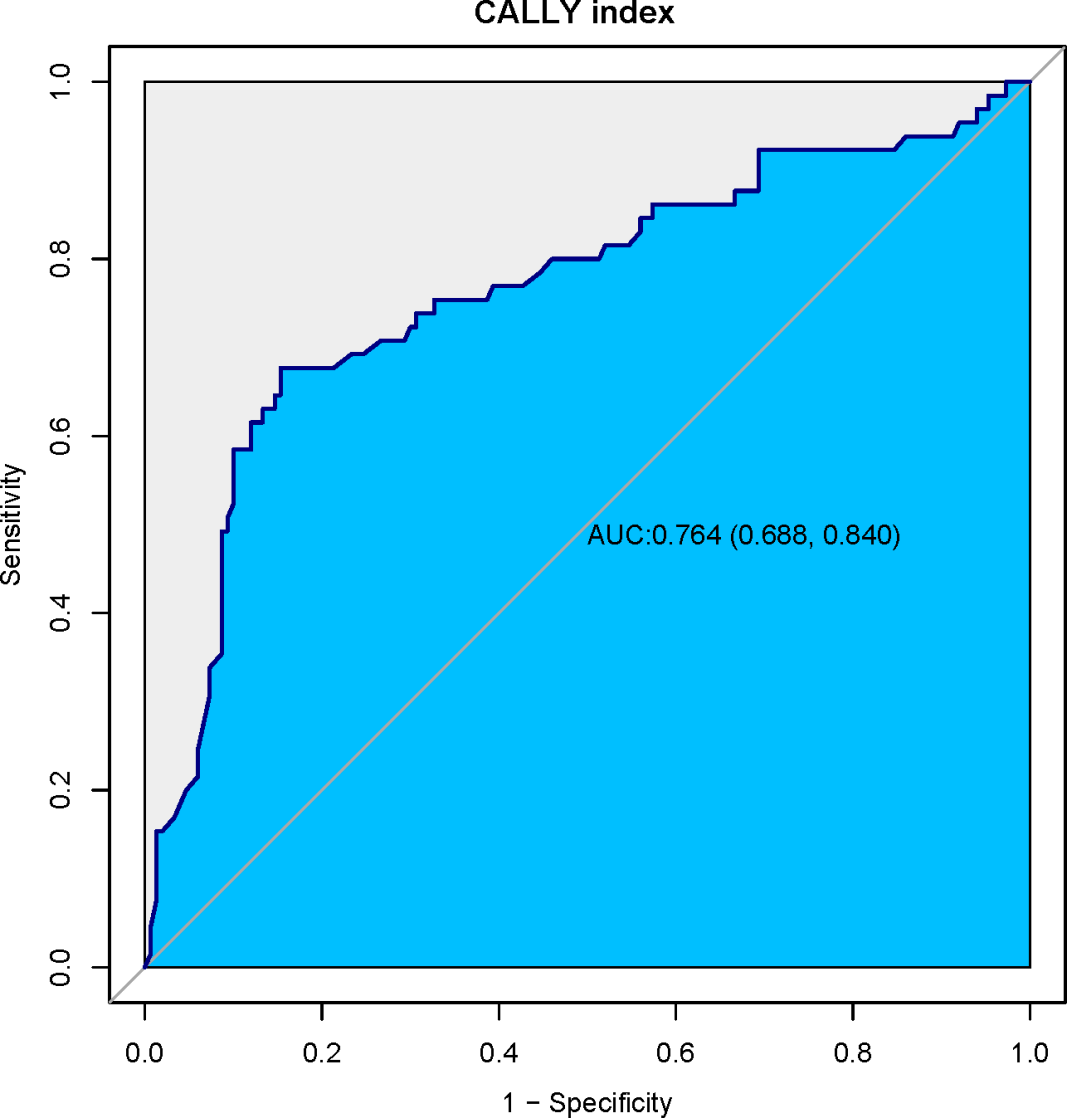
Receiver operating characteristic (ROC) curve for the CALLY index. This ROC curve illustrates the diagnostic performance of the CALLY index in predicting postoperative pneumonia in patients with resectable esophageal squamous cell carcinoma (ESCC). With an area under the curve (AUC) of 0.764 (95% CI: 0.688, 0.840; p < 0.001), the CALLY index demonstrates strong discriminatory ability. The optimal cutoff value of 1.97, identified via the Youden index, yields a sensitivity of 67.69% and a specificity of 84.67%.

The prognostic ability of the CALLY index for predicting postoperative pneumonia was assessed using the receiver operating characteristic (ROC) curve analysis. As depicted in **Figure 2**, the area under the curve (AUC) for the CALLY index was 0.764 (95% CI: 0.688, 0.840; p < 0.001). This indicates a good discriminatory ability of the CALLY index in predicting postoperative pneumonia outcomes in patients with resectable esophageal squamous cell carcinoma (ESCC). The optimal cutoff value for the CALLY index, obtained by maximizing the Youden index, was identified as 1.97. At the optimal cutoff value, the sensitivity was 67.69%, specificity was 84.67%, negative predictive value (NPV) was 85.81%, and positive predictive value (PPV) was 65.67%. The overall accuracy of the CALLY index in predicting postoperative pneumonia occurrence is now reported at 76.14%.

### 3.2 Demographic and clinical characteristics of the patient cohort

The cohort comprised 215 patients with resectable esophageal squamous cell carcinoma, whose characteristics were initially differentiated into High-CALLY and Low-CALLY groups before propensity score matching (PSM). As illustrated in **Table 1**, gender distribution, mean age, body mass index (BMI), and pulmonary function tests (FEV1 and FEV1/FVC%) showed no statistically significant differences between the High-CALLY (n = 166) and Low-CALLY (n = 49) groups (p > 0.05). Significant disparities were evident in smoking habits (p = 0.01) and the CALLY index (p < 0.001), underscoring distinct inflammatory status across patient subsets. Importantly, the incidence of postoperative pneumonia was significantly higher in the Low-CALLY group (69.4%) than in the High-CALLY group (18.7%) (p < 0.001), highlighting the relevance of the CALLY index as a potential predictor. To mitigate confounding biases and establish a more balanced comparison, propensity score matching (PSM) was employed. Post-PSM (**Table 2**), 88 patients—44 in each group—were matched to equalize baseline characteristics, significantly enhancing group comparability. The application of PSM elucidated no significant differences in gender, age, BMI, pulmonary function parameters, tumor location, and other clinicopathological variables, ensuring rigorous examination of the CALLY index’s prognostic capability (p > 0.05 across most variables). The balance achieved through PSM underscores its efficacy in harmonizing baseline discrepancies, reinforcing the robustness of the subsequent analyses.

**Table 1:** Baseline characteristics and outcomes of participants before propensity score matching.

Characteristics	Total (n = 215)	CALLY index		P value
		High-CALLY cohort (n = 166)	Low-CALLY cohort (n = 49)	
Gender, n (%)				0.883
Female	64 (29.8)	49 (29.5)	15 (30.6)	
Male	151 (70.2)	117 (70.5)	34 (69.4)	
Age (year), Mean ± SD	65.7 ± 6.8	65.3 ± 7.0	66.9 ± 5.9	0.148
BMI (kg/m^2^), Mean ± SD	22.6 ± 3.2	22.4 ± 3.2	23.2 ± 3.2	0.122
FEV1, Mean ± SD	91.0 ± 18.8	91.3 ± 18.8	90.0 ± 18.8	0.672
FEV1/FVC%, Mean ± SD	94.5 ± 13.0	94.0 ± 13.3	96.1 ± 12.2	0.314
Hypertension, n (%)				0.736
No	149 (69.3)	116 (69.9)	33 (67.3)	
Yes	66 (30.7)	50 (30.1)	16 (32.7)	
Diabetes mellitus, n (%)				0.229
No	198 (92.1)	155 (93.4)	43 (87.8)	
Yes	17 ( 7.9)	11 (6.6)	6 (12.2)	
Smoking, n (%)				0.01
No	154 (71.6)	126 (75.9)	28 (57.1)	
Yes	61 (28.4)	40 (24.1)	21 (42.9)	
Tumor location, n (%)				0.784
Upper	35 (16.3)	28 (16.9)	7 (14.3)	
Middle	118 (54.9)	89 (53.6)	29 (59.2)	
Low	62 (28.8)	49 (29.5)	13 (26.5)	
Tumor grade, n (%)				0.104
High	44 (20.5)	38 (22.9)	6 (12.2)	
Moderate	121 (56.3)	94 (56.6)	27 (55.1)	
Low	50 (23.3)	34 (20.5)	16 (32.7)	
TNM, n (%)				0.062
I	118 (54.9)	94 (56.6)	24 (49)	
II	39 (18.1)	27 (16.3)	12 (24.5)	
IIIa	43 (20.0)	37 (22.3)	6 (12.2)	
IIIb	15 ( 7.0)	8 (4.8)	7 (14.3)	
pT, n (%)				0.557
T1	61 (28.4)	50 (30.1)	11 (22.4)	
T2	100 (46.5)	76 (45.8)	24 (49)	
T3	54 (25.1)	40 (24.1)	14 (28.6)	
pN, n (%)				0.832
N0	150 (69.8)	117 (70.5)	33 (67.3)	
N1	50 (23.3)	37 (22.3)	13 (26.5)	
N2	15 ( 7.0)	12 (7.2)	3 (6.1)	
ASA, n (%)				0.564
1	139 (64.7)	109 (65.7)	30 (61.2)	
2	54 (25.1)	39 (23.5)	15 (30.6)	
3	22 (10.2)	18 (10.8)	4 (8.2)	
CALLY index, Mean ± SD	4.1 ± 1.9	5.2 ± 1.2	1.6 ± 0.4	< 0.001
Pneumonia, n (%)				< 0.001
No	150 (69.8)	135 (81.3)	15 (30.6)	
Yes	65 (30.2)	31 (18.7)	34 (69.4)	
Esophagocutaneous fistula, n (%)				0.66
No	208 (96.7)	161 (97)	47 (95.9)	
Yes	7 ( 3.3)	5 (3)	2 (4.1)	
Length of hospital stay, Mean ± SD	14.2 ± 8.2	13.7 ± 7.3	16.0 ± 10.4	0.088
Microvascular invasion, n (%)				0.248
No	142 (66.0)	113 (68.1)	29 (59.2)	
Yes	73 (34.0)	53 (31.9)	20 (40.8)	

BMI, Body Mass Index; FEV1, Forced Expiratory Volume in the first second; FVC, Forced Vital Capacity; TNM, Tumor, Node, Metastasis; ASA, American Society of Anesthesiologists; SD, Standard Deviation.

**Table 2:** Baseline characteristics and outcomes of participants after propensity score matching.

Characteristics	Total (n = 88)	CALLY index		P value
		High-CALLY cohort (n = 44)	Low-CALLY cohort (n = 44)	
Gender, n (%)				0.821
Female	29 (33.0)	14 (31.8)	15 (34.1)	
Male	59 (67.0)	30 (68.2)	29 (65.9)	
Age (year), Mean ± SD	66.4 ± 6.6	66.3 ± 7.3	66.4 ± 6.0	0.987
BMI (kg/m^2^), Mean ± SD	23.4 ± 3.4	23.7 ± 3.8	23.1 ± 3.1	0.378
FEV1, Mean ± SD	89.7 ± 18.7	89.7 ± 20.2	89.7 ± 17.3	0.986
FEV1/FVC%, Mean ± SD	96.4 ± 11.7	97.5 ± 12.4	95.3 ± 11.0	0.38
Hypertension, n (%)				0.509
No	55 (62.5)	26 (59.1)	29 (65.9)	
Yes	33 (37.5)	18 (40.9)	15 (34.1)	
Diabetes mellitus, n (%)				0.747
No	77 (87.5)	39 (88.6)	38 (86.4)	
Yes	11 (12.5)	5 (11.4)	6 (13.6)	
Smoking, n (%)				1
No	54 (61.4)	27 (61.4)	27 (61.4)	
Yes	34 (38.6)	17 (38.6)	17 (38.6)	
Tumor location, n (%)				0.886
Upper	13 (14.8)	6 (13.6)	7 (15.9)	
Middle	47 (53.4)	23 (52.3)	24 (54.5)	
Low	28 (31.8)	15 (34.1)	13 (29.5)	
Tumor grade, n (%)				0.65
High	10 (11.4)	4 (9.1)	6 (13.6)	
Moderate	54 (61.4)	29 (65.9)	25 (56.8)	
Low	24 (27.3)	11 (25)	13 (29.5)	
TNM, n (%)				0.886
I	38 (43.2)	19 (43.2)	19 (43.2)	
II	36 (40.9)	19 (43.2)	17 (38.6)	
IIIa	8 ( 9.1)	4 (9.1)	4 (9.1)	
IIIb	6 ( 6.8)	2 (4.5)	4 (9.1)	
pT, n (%)				0.97
T1	20 (22.7)	10 (22.7)	10 (22.7)	
T2	41 (46.6)	20 (45.5)	21 (47.7)	
T3	27 (30.7)	14 (31.8)	13 (29.5)	
pN, n (%)				1
N0	62 (70.5)	31 (70.5)	31 (70.5)	
N1	25 (28.4)	13 (29.5)	12 (27.3)	
N2	1 ( 1.1)	0 (0)	1 (2.3)	
ASA, n (%)				0.832
1	54 (61.4)	28 (63.6)	26 (59.1)	
2	28 (31.8)	14 (31.8)	14 (31.8)	
3	6 ( 6.8)	2 (4.5)	4 (9.1)	
CALLY index, Mean ± SD	3.5 ± 3.0	5.8 ± 2.7	1.3 ± 0.4	< 0.001
Pneumonia, n (%)				< 0.001
No	42 (47.7)	29 (65.9)	13 (29.5)	
Yes	46 (52.3)	15 (34.1)	31 (70.5)	
Esophagocutaneous fistula, n (%)				1
No	85 (96.6)	43 (97.7)	42 (95.5)	
Yes	3 ( 3.4)	1 (2.3)	2 (4.5)	
Length of hospital stay, Mean ± SD				0.665
Microvascular invasion, n (%)	52 (59.1)	27 (61.4)	25 (56.8)	
No	36 (40.9)	17 (38.6)	19 (43.2)	
Yes	15.7 ± 10.4	15.9 ± 11.1	15.5 ± 9.7	0.846

BMI, Body Mass Index; FEV1, Forced Expiratory Volume in the first second; FVC, Forced Vital Capacity; TNM, Tumor, Node, Metastasis; ASA, American Society of Anesthesiologists; SD, Standard Deviation.

### 3.3 Univariate and multivariate analysis of risk factors for postoperative pneumonia

As presented in **Table 3**, before applying PSM, several clinicopathological factors were assessed for their association with postoperative pneumonia. Univariate analysis identified age, TNM stage, tumor grade, esophagocutaneous fistula presence, and length of hospital stay as significant predictors of postoperative pneumonia. Notably, the CALLY index demonstrated a strong protective effect against the occurrence of postoperative pneumonia (OR = 0.64; 95% CI: 0.54-0.76; p < 0.001). In the subsequent multivariate analysis, the CALLY index (adjusted OR = 0.66; 95% CI: 0.54-0.79; p < 0.001), age (adjusted OR = 1.06; 95% CI: 1-1.12; p = 0.041), and TNM IIIb stage (adjusted OR = 4.33; 95% CI: 1-18.69; p = 0.049) emerged as independent predictors of postoperative pneumonia, underscoring the complex interplay between demographic factors, tumor characteristics, and the inflammatory profile captured by the CALLY index. Following PSM to attain balanced baseline characteristics (see **Table 4**), logistic regression analyses were re-conducted to ensure robustness in the identified associations. Post-PSM analysis yielded similar insights, albeit with some variations in significance levels. The univariate examination post-PSM continued to highlight the CALLY index (OR = 0.65; 95% CI: 0.52-0.82; p < 0.001) and length of hospital stay (OR = 1.22; 95% CI: 1.08-1.37; p = 0.001) as significant factors. Upon further adjustment in multivariate regression, the CALLY index remained a protective factor against postoperative pneumonia, with an adjusted OR reduced to 0.57 (95% CI: 0.41-0.79; p = 0.001). Additionally, length of hospital stay (adjusted OR = 1.21; 95% CI: 1.08-1.36; p = 0.001) retained its role as a risk factor, while other variables did not achieve statistical significance. Overall, both pre- and post-PSM analyses reinforce the CALLY index as a pivotal independent predictor, suggesting its substantial utility in forecasting and potentially mitigating the risk of postoperative pneumonia in patients with resectable esophageal squamous cell carcinoma.

**Table 3:** The results of univariate and multivariate logistic analyses, along with the predictors of postoperative pneumonia prior to propensity score matching.

Clinical variables	Postoperative pneumonia			
	crude.OR (95%CI)	crude.P value	adj.OR (95%CI)	adj.P value
Gender				
Female	Ref		Ref	
Male	1.04 (0.55~1.96)	0.91		
Age	1.08 (1.03~1.13)	0.002	1.06 (1~1.12)	0.041
BMI	1 (0.92~1.1)	0.929		
FEV1/FVC%	1.01 (0.99~1.03)	0.329		
Hypertension				
No	Ref			
Yes	1.11 (0.6~2.08)	0.736		
Diabetes mellitus				
No	Ref			
Yes	2.2 (0.81~5.98)	0.123		
Smoking				
No	Ref			
Yes	1.61 (0.86~3.02)	0.135		
Tumor location				
Upper	Ref			
Middle	1.54 (0.64~3.72)	0.335		
Low	1.61 (0.62~4.16)	0.329		
Tumor grade				
High	Ref			
Middle	1.91 (0.81~4.5)	0.141		
Low	3.26 (1.26~8.43)	0.015		
TNM				
I	Ref			
II	2.6 (1.21~5.6)	0.014	1.62 (0.6~4.38)	0.346
IIIa	1.46 (0.67~3.19)	0.341	2.61 (1.01~6.73)	0.047
IIIb	3.85 (1.28~11.59)	0.016	4.33 (1~18.69)	0.049
ASA Classification				
1	Ref			
2	0.88 (0.44~1.74)	0.713		
3	0.46 (0.15~1.45)	0.187		
CALLY index	0.64 (0.54~0.76)	<0.001	0.66 (0.54~0.79)	<0.001
Esophagocutaneous fistula				
No	Ref		Ref	
Yes	6.17 (1.16~32.66)	0.032	1.08 (0.16~7.48)	0.939
Length of hospital stay	1.14 (1.07~1.21)	<0.001	1.14 (1.07~1.22)	<0.001
Microvascular invasion				
No	Ref			
Yes	1.61 (0.88~2.94)	0.124		

BMI, Body Mass Index; FEV1, Forced Expiratory Volume in the first second; FVC, Forced Vital Capacity; TNM, Tumor, Node, Metastasis; ASA, American Society of Anesthesiologists; OR, Odds Ratio; CI, Confidence Interval.

**Table 4:** The results of univariate and multivariate logistic analyses, along with the predictors of postoperative pneumonia following propensity score matching.

Clinical variables	Postoperative pneumonia			
	crude.OR (95%CI)	crude.P value	adj.OR (95%CI)	adj.P value
Gender				
Female	Ref			
Male	1.93 (0.78~4.75)	0.154		
Age	1.07 (1~1.15)	0.056	1.06 (0.97~1.15)	0.232
BMI	0.95 (0.84~1.08)	0.437		
FEV1/FVC%	1.01 (0.97~1.05)	0.561		
Hypertension				
No	Ref			
Yes	0.78 (0.33~1.86)	0.582		
Diabetes mellitus				
No	Ref			
Yes	1.11 (0.31~3.95)	0.872		
Smoking				
No	Ref			
Yes	0.71 (0.3~1.68)	0.438		
Tumor location				
Upper	Ref			
Middle	1.33 (0.39~4.54)	0.654		
Low	1.35 (0.36~5.04)	0.659		
Tumor grade				
High	Ref			
Middle	0.53 (0.13~2.11)	0.37		
Low	1.33 (0.29~6.12)	0.711		
TNM				
I	Ref			
II	1.56 (0.62~3.9)	0.346		
IIIa	0.67 (0.14~3.19)	0.612		
IIIb	2.22 (0.36~13.62)	0.388		
ASA Classification				
1	Ref			
2	0.44 (0.18~1.13)	0.089		
3	0.69 (0.13~3.73)	0.664		
CALLY	0.65 (0.52~0.82)	<0.001	0.57 (0.41~0.79)	0.001
Esophagocutaneous fistula				
No	Ref			
Yes	4.15 (0.78~6.19)	0.99		
Length of hospital stay	1.22 (1.08~1.37)	0.001	1.21 (1.08~1.36)	0.001
Microvascular invasion				
No	Ref			
Yes	2.73 (1.13~6.61)	0.026		

BMI, Body Mass Index; FEV1, Forced Expiratory Volume in the first second; FVC, Forced Vital Capacity; TNM, Tumor, Node, Metastasis; ASA, American Society of Anesthesiologists; OR, Odds Ratio; CI, Confidence Interval.

### 3.4 Curve fitting analysis of logistic regression for the CALLY index, length of hospital stay, and risk of postoperative pneumonia

**Figure 3:**
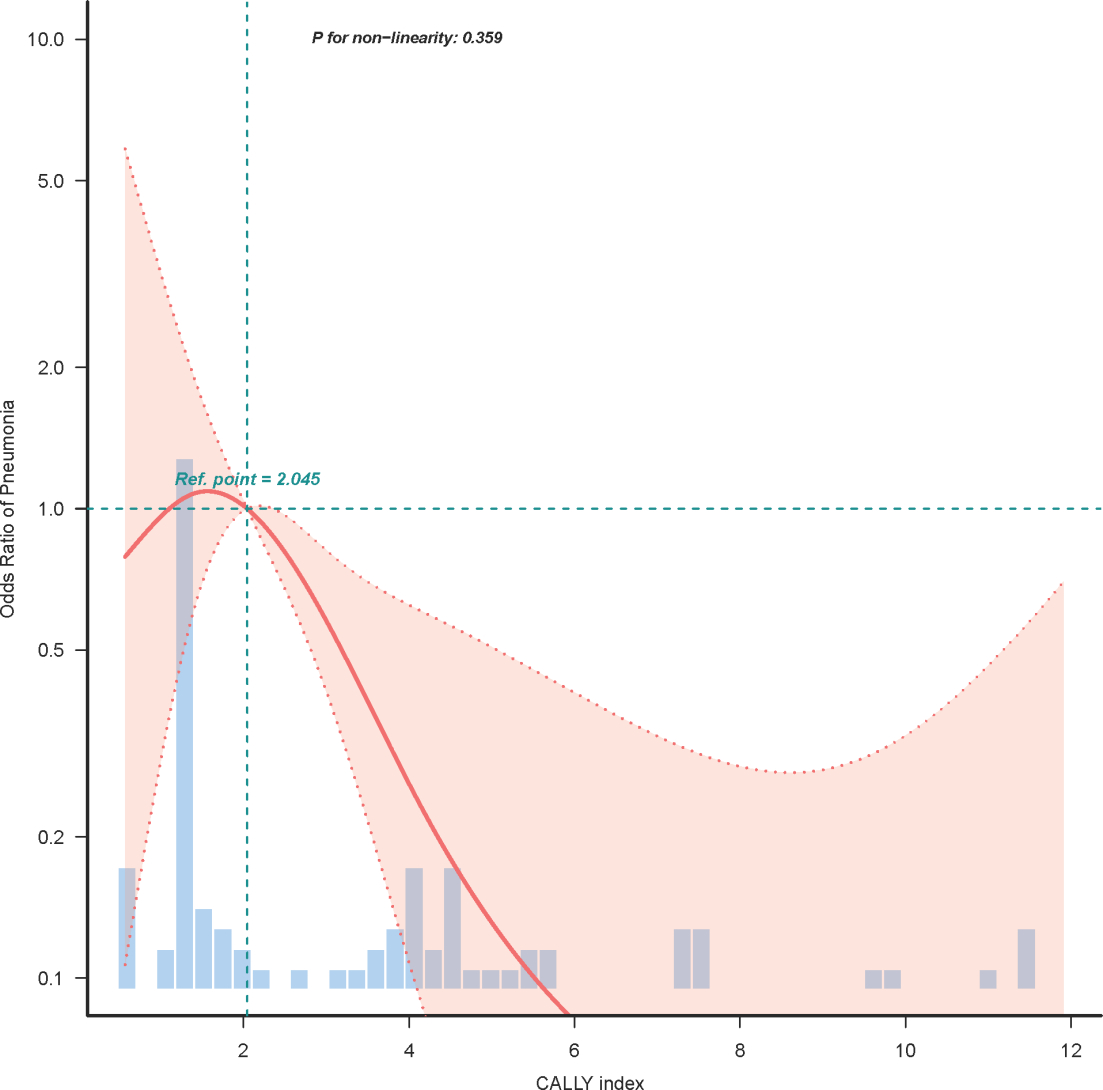
Logistic regression curve analysis of CALLY index and postoperative pneumonia risk. This figure displays the non-linear relationship between the CALLY index and the odds ratio for postoperative pneumonia, analyzed through logistic regression curve fitting after propensity score matching (PSM). The plot highlights that lower CALLY index scores are associated with increased pneumonia risk, with a reference inflection point observed at a CALLY index of 2.045. The histogram below the curve represents the distribution of patients across varying CALLY index levels.

**Figure 4:**
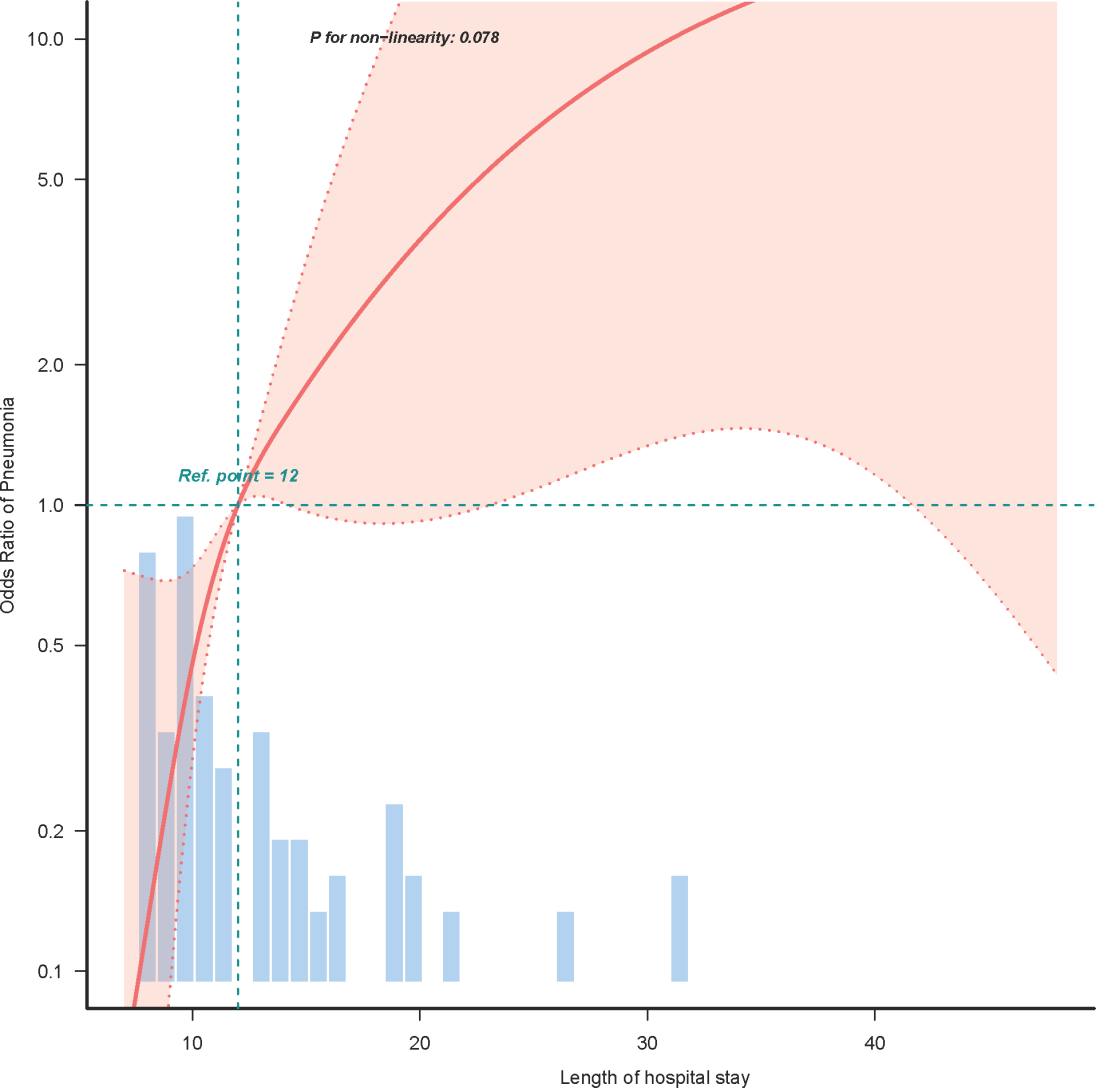
Curve fitting analysis of the length of hospital stay and the risk of postoperative pneumonia. The figure illustrates the relationship between the length of hospital stay and the odds ratio of developing postoperative pneumonia in patients with resectable esophageal squamous cell carcinoma (ESCC). A logistic regression curve fitting model was applied to explore this association, revealing a significant relationship (P for non-linearity = 0.078) after propensity score matching (PSM). The curve indicates that the risk of postoperative pneumonia increases substantially when the hospital stay exceeds 12 days, emphasizing the need for strategies to minimize hospital duration. The histogram below the curve reflects the distribution of patient hospital stays.

In our endeavor to characterize the relationship between the CALLY index and postoperative pneumonia, logistic regression curve fitting analysis was employed. **Figure 3** illustrates the relationship between the CALLY index and the odds ratio of postoperative pneumonia after propensity score matching (PSM). The analysis reveals a non-linear association (P for non-linearity = 0.359), indicating that lower CALLY index scores are correlated with a higher risk of postoperative pneumonia. A reference point is observed at a CALLY index of 2.045, beyond which the risk decreases. The histogram beneath the curve visualizes the patient distribution across different CALLY index values, emphasizing this relationship. Additionally, **Figure 4** examines the relationship between the length of hospital stay and the odds of developing postoperative pneumonia. Here, a significant relationship is evidenced (P for non-linearity = 0.078), with the risk increasing substantially when the hospital stay exceeds 12 days. This trend underscores the importance of minimizing hospital stay to reduce pneumonia risk.

## 4 Discussion

In our study of 215 ESCC patients, the CALLY index emerged as a pivotal preoperative indicator of postoperative pneumonia risk. Utilizing ROC curve analysis, the CALLY index demonstrated a strong discriminatory ability with an area under the curve (AUC) of 0.764, surpassing conventional parameters traditionally used in clinical assessment. With a cutoff value of 1.97 identified, this index offers substantial sensitivity and specificity, laying the groundwork for its integration as a predictive tool in surgical oncology. Upon multivariate analysis, the CALLY index consistently revealed its robustness as an independent protective factor against postoperative pneumonia. Our findings suggest that lower CALLY index values correlate with heightened pneumonia risk, underscoring the importance of preoperative nutritional and inflammatory status. These insights are particularly salient given the significant rates of postoperative pneumonia observed in the Low-CALLY cohort compared to the High-CALLY cohort, both pre- and post-propensity score matching (PSM). Our analysis highlights that the inclusion of variables such as age, TNM stage, and length of hospital stay continues to be influential. Notably, patients with lower CALLY index scores experienced extended hospital stays, further exacerbating pneumonia risk. The combined logistic regression and curve fitting analyses provided a nuanced view of the nonlinear relationship between CALLY index values and pneumonia risk, indicating a crucial inflection point aiding clinical interpretation. Our study, therefore, positions the CALLY index as a promising marker that integrates nutritional, immune, and inflammatory statuses, offering a comprehensive tool to guide clinical decision-making. This could facilitate tailored preoperative interventions aimed at optimizing patient condition and potentially decreasing postoperative complications.

The CALLY index integrates markers such as albumin, lymphocyte count, and C-reactive protein (CRP), which collectively reflect nutritional status and systemic inflammation. Low albumin levels indicate inadequate nutritional reserves, potentially delaying recovery and compromising immune responses. Lymphocytes play a crucial role in immune surveillance; their decrease suggests weakened immune defenses, while elevated CRP levels indicate increased inflammatory activity, further heightening the risk of infections such as pneumonia. Our study reveals that the correlation between lower CALLY index scores and increased pneumonia risk aligns with theoretical expectations based on the physiology of inflammatory responses. The components of the index effectively capture the systemic inflammation and immune compromise associated with the pathophysiological mechanisms leading to pneumonia. Prior research has established a profound link between the CALLY index and survival outcomes in various cancers such as gastric, colorectal, and non-small cell lung cancer. For instance, research conducted by Fukushima and colleagues identified that a preoperative CALLY index below the threshold of 2 profoundly marked a decreased overall and relapse-free survival after surgery for gastric cancer, highlighting its predictive power for unfavorable clinical outcomes (32). In colorectal cancer, investigations by Takeda et al. marked a CALLY index score below 2 as an independent predictor for superior disease-free survival rates (33). Moreover, Liu and associates’ work in non-small cell lung cancer revealed the CALLY index’s efficacy through a prognostic nomogram that achieved a noteworthy C-index of 0.697 for predicting overall survival rates (31). Expanding on these contributions, recent research by Wang et al. demonstrated that setting a cut-off value at 3 for the CALLY index effectively stratified patients into distinct prognostic categories, with those having an index of 3 or above exhibiting notably enhanced survival outcomes in the studied populations. Both univariate and multivariate Cox regression analyses corroborated the CALLY index as a prognostic determinant for overall survival (OS) and disease-free survival (DFS), reinforcing its stature as a predictor of disease trajectory and long-term prognosis in malignant tumors (34). Despite its recognized value, the literature examining the CALLY index’s role in esophageal squamous cell carcinoma is relatively underexplored (35). Our current research enriches this domain by shedding light on the index’s implications for postoperative care in esophageal squamous cell carcinoma patients. The early detection of the CALLY index parameters could facilitate personalized prognostic assessments and refine therapeutic strategies, underscoring its utility in clinical decision-making and patient management.

The prognostic landscape for cancer patients is increasingly recognized as being influenced by the interplay of nutritional status, immune-inflammatory mechanisms, and the inherent biology of the cancer itself. Serum albumin (ALB) levels, a parameter frequently utilized as an indicator of nutritional well-being, have significant ties to patient prognoses in oncological contexts (36). Various inflammatory mediators such as tumor necrosis factor (TNF), which augments microvascular leakiness, along with interleukin-1 (IL-1) and interleukin-6 (IL-6), known to suppress albumin production, contribute to the observed decrease in serum albumin among cancer-afflicted individuals (37, 38). In parallel, serum C-reactive protein (CRP) is a well-established symbol of systemic inflammation, promoting the liberation of pro-inflammatory cytokines like IL-1, IL-6, and TNF-α (39, 40). Such an inflammatory storm can lead to significant protein depletion, ultimately relating to increased mortality rates within the cancer patient population. Elevated CRP concentrations have been linked with more progressed cancer stages as per the TNM classification, indicating a magnified inflammatory reaction within the disease state. Furthermore, lymphocytes (LYMs) act as harbingers of immune system potency and partake actively in the tumor milieu, constraining tumor cell growth and spread (41). In the nexus of cancer pathophysiology, nutrition, and inflammation stands the CALLY index, potentially eclipsing classical prognostic indices in efficacy (42). This composite metric, accounting for serum parameters such as albumin, lymphocyte count, and CRP concentrations, blends key insights on a patient’s nutritive state, immunological fortitude, and inflammatory milieu (29). For instance, depleted albumin levels may be emblematic of malnutrition, detracting from post-surgical recovery potential (43). A subdued lymphocyte count hints at a compromised immunological apparatus that could diminish the organism’s pathogen defense (44). Conversely, heightened CRP identifies acute inflammation, providing a window into the body’s systemic inflammatory activity (45). Conclusively, the CALLY index operates as an encompassing prognostic gauge for postoperative infection risks, establishing a bridge between the oncological microenvironment and overarching physiological status. Through its integration of salient health parameters, the index affords a multifaceted perspective aiding in the prognostication and management of cancer-related outcomes.

This research offers insightful contributions regarding the CALLY index’s applicability as a predictor for postoperative respiratory complications in patients with esophageal squamous cell carcinoma. However, it is important to acknowledge certain inherent limitations of the study. One limitation of this study is the exclusion of patients undergoing neoadjuvant treatment, which potentially limits the CALLY index’s applicability in broader clinical scenarios where such treatments are prevalent. Future studies should consider including this cohort to explore whether preoperative treatments correlate with worse CALLY scores and increased pneumonia risk. Additionally, the retrospective methodology employed may introduce selection biases. Moreover, the findings are derived from a single institution’s patient cohort, potentially affecting the broadness of their applicability. To establish the CALLY index’s reliability and relevance across diverse clinical settings, prospective studies that encompass multiple centers are warranted in the future to confirm our observations.

## 5 Conclusion

This retrospective cohort study underscores the predictive capability of the CALLY index in foreseeing postoperative pneumonia among patients with resectable ESCC, indicating its value as a predictive tool for enhancing perioperative risk assessment and management. Our findings reveal that a lower preoperative CALLY index score is significantly associated with an increased risk of postoperative pneumonia. Through rigorous ROC curve analysis, the CALLY index demonstrated substantial discriminatory power with an AUC of 0.764, offering a reliable sensitivity and specificity with a cutoff value of 1.97. Multivariate analyses further affirmed its role as an independent predictor, with lower scores correlating with heightened pneumonia risk, irrespective of other clinical factors.

This retrospective cohort study underscores the predictive capability of the CALLY index in foreseeing postoperative pneumonia among patients with resectable ESCC, indicating its value as a predictive tool for enhancing perioperative risk assessment and management.

## Data Availability

The raw data supporting the conclusions of this article will be made available by the authors, without undue reservation.
